# Transcriptome Analysis of Green Peach Aphid (*Myzus persicae*): Insight into Developmental Regulation and Inter-Species Divergence

**DOI:** 10.3389/fpls.2016.01562

**Published:** 2016-10-21

**Authors:** Rui Ji, Yujun Wang, Yanbin Cheng, Meiping Zhang, Hong-Bin Zhang, Li Zhu, Jichao Fang, Keyan Zhu-Salzman

**Affiliations:** ^1^Institute of Plant Protection, Jiangsu Academy of Agricultural SciencesNanjing, China; ^2^Department of Entomology, Texas A&M UniversityCollege Station, TX, USA; ^3^Ministry of Agriculture Key Laboratory of Agricultural Entomology, Institute of Insect Sciences, Zhejiang UniversityHangzhou, China; ^4^Department of Plant Pathology and Microbiology, Texas A&M UniversityCollege Station, TX, USA; ^5^Department of Soil and Crop Sciences, Texas A&M UniversityCollege Station, TX, USA; ^6^Biotechnology Research Institute, Chinese Academy of Agricultural SciencesBeijing, China

**Keywords:** *Myzus persicae*, *Acyrthosiphon pisum*, nymph and adult, transcriptome, developmental regulation, synonymous and nonsynonymous substitutions, host plant adaptation

## Abstract

Green peach aphid (*Myzus persicae*) and pea aphid (*Acyrthosiphon pisum*) are two phylogenetically closely related agricultural pests. While pea aphid is restricted to Fabaceae, green peach aphid feeds on hundreds of plant species from more than 40 families. Transcriptome comparison could shed light on the genetic factors underlying the difference in host range between the two species. Furthermore, a large scale study contrasting gene expression between immature nymphs and fully developed adult aphids would fill a previous knowledge gap. Here, we obtained transcriptomic sequences of green peach aphid nymphs and adults, respectively, using Illumina sequencing technology. A total of 2244 genes were found to be differentially expressed between the two developmental stages, many of which were associated with detoxification, hormone production, cuticle formation, metabolism, food digestion, and absorption. When searched against publically available pea aphid mRNA sequences, 13,752 unigenes were found to have no homologous counterparts. Interestingly, many of these unigenes that could be annotated in other databases were involved in the “xenobiotics biodegradation and metabolism” pathway, suggesting the two aphids differ in their adaptation to secondary metabolites of host plants. Conversely, 3989 orthologous gene pairs between the two species were subjected to calculations of synonymous and nonsynonymous substitutions, and 148 of the genes potentially evolved in response to positive selection. Some of these genes were predicted to be associated with insect-plant interactions. Our study has revealed certain molecular events related to aphid development, and provided some insight into biological variations in two aphid species, possibly as a result of host plant adaptation.

## Introduction

Aphids (Insecta: Hemiptera), a group of economically important insect pests that consume plant phloem sap, cause substantial losses of crop yield by direct feeding on host plants and by vectoring plant viruses (Dixon, 1998). More than 450 species within Aphididae attack agricultural and horticultural plants, of which over 100 are categorized as significant and economically important pests (Blackman and Eastop, 1984). While some aphids are specific to plant species in a single taxonomic family, others have an exceptionally broad host range across many plant families. Green peach aphid (*Myzus persicae*) is a generalist with a host range comprising 40 different plant families including Brassicaceae, Solanaceae, and Fabaceae. Moreover, it is the most versatile viral vector, capable of transmitting more than 100 plant viruses (Ramsey et al., 2007). In contrast, pea aphid (*Acyrthosiphon pisum*) feeds specifically on legumes. Despite different feeding habits, they are both classified in the tribe Macrosiphini within the subfamily Aphidinae (von Dohlen et al., 2006). The close relationship between the two aphids is further supported by analysis of mitochondrial and nuclear sequences as well as transcriptomic sequence comparisons (Ramsey et al., 2007; Kim and Lee, 2008). Due to the difference in host range, green peach aphids most likely ingest toxic metabolites that pea aphids would not normally encounter, such as glucosinolates in Brassicaceae and alkaloids in Solanaceae, necessitating a more complex metabolic system (Ramsey et al., 2010).

Hemipteran immature nymphs and fully developed adults sometimes differ in their feeding behavior. *Lygus hesperus* nymphs prefer developing cotton squares, whereas adults prefer vegetative structures (Snodgrass, 1998). In three spittlebug species (*Aeneolamia varia, A. reducta* and *Zulia carbonaria*), foliage-feeding adults are more capable of feeding upon resistant hybrid crops than root- and stem-feeding nymphs (Cardona et al., 2010). Besides host and tissue preferences, quantity of food intake can vary (Banks and Macaulay, 1965). Profiling in nymphal and adult transcriptomes could reveal biological properties that are developmental stage-specific. In Asian citrus psyllid (*Diaphorina citri*) for instance, the transcriptome comparison revealed distinct patterns of protein and energy requirements between nymphs and adults (Vyas et al., 2015). This approach has also identified differentially expressed resistance/detoxification genes, e.g., cytochrome P450, glutathione S-transferase (GST), and ATP-binding cassette transporter genes from two developmental stages of a thiamethoxam-resistant strain of whitefly (Yang et al., 2013). Contrasting gene expression among different insect developmental stages on a large scale can not only shed light on development modulation, reproduction, and developmental stage-specific interaction with host plant, xenobiotics, and invading microbes, but can also facilitate the improvement of pest management strategies (Yang et al., 2013; Tian et al., 2015; Vyas et al., 2015). However, stage-specific gene expression in immature nymphs and fully developed adults has not yet been characterized in aphids.

While comparative genomic sequence analysis has furnished tremendous information regarding genetic factors underlying inter-species divergence (Chinwalla et al., 2002; Kaufman et al., 2002; Kirkness et al., 2003; Zdobnov and Bork, 2007; Arensburger et al., 2010; Bonasio et al., 2010; Werren et al., 2010), an increasing number of studies have applied RNA-seq for this purpose, particularly in species whose genome sequences are unavailable. For example, transcriptomic comparisons have been performed between different aphids, *A. pisum* vs. *Sitobion avenae* (Wang et al., 2014), whitefly (*Bemisia tabaci*) species complexes Middle East-Asia Minor 1 vs. Mediterranean (Wang et al., 2011), ranid frogs *Rana chensinensis* vs. *Rana kukunoris* (Yang et al., 2012), ornamental primrose species *Primula poissonii* vs. *Primula wilsonii* (Zhang L. et al., 2013), and fishes, *Erythroculter ilishaeformis* vs. *Danio rerio* (Ren et al., 2014). Comparisons among pea aphid, green peach aphid and grain aphid *(S. avenae)* have enabled investigation of the transcriptome evolution and understanding of the differences in host plant adaptation and insecticide resistance among them (Ollivier et al., 2010; Ramsey et al., 2010; Wang et al., 2014). Between grain aphid and pea aphid 340 gene orthologs are considered to be under positive selection based on the rates of nonsynonymous (Ka) and synonymous (Ks) substitutions (Wang et al., 2014). Such orthologs were also identified when Ollivier et al. (2010) compared coding sequences (CDSs) derived from the genome sequence of pea aphid and EST database derived from 5 tissues of green peach aphids reared on 5 host plants (Ramsey et al., 2007). Later, Ramsey et al. (2010) sequenced the transcriptome from mixed stages of green peach aphids using 454 pyrosequencing. Besides the reads mapped to the existing ESTs, they obtained 47,832 additional unigenes with a mean length of 160 bp, from which they identified more detoxification genes in green peach aphid than in pea aphid (Ramsey et al., 2010). However, limited transcriptomic information may not fully reflect the divergence between the two species.

In this study, we performed transcriptomic sequencing of green peach aphid nymphs and adults using Illumina RNA-seq technology, *de novo* assembled sequencing reads, and annotated the resulting unigenes. Gene expression profiling between nymphs and adults identified genes potentially involved in development modulation. Furthermore, comparative transcriptomic analyses identified genes unique to green peach aphid (relative to pea aphid) and orthologous gene pairs under positive selection. Data analysis has helped expose certain genetic factors underlying host plant adaptation by the two destructive aphid species.

## Materials and methods

### Plant growth and insect rearing

Arabidopsis ecotype Col-0 plants were grown in LP5 potting medium (Sun Gro Horticulture, Agawam, WA, USA) in an environmental chamber at 23⋅C (day)/21⋅C (night), 65% relative humidity (RH), and a photosynthetic photon flux density of 88 μmol m^−2^ s^−1^ with a 12-h light/12-h dark photoperiod. The green peach aphid (a tobacco-adapted red lineage from Dr. Georg Jander, Boyce Thompson Institute for Plant Research, Cornell University) had been maintained on Col-0 for over 40 generations. Age-synchronized nymphs and adults were subjected to RNA extraction as described below.

### RNA isolation and transcriptome sequencing

Neonate nymphs (within 16 h) were placed on 4-week-old Col-0 plants for 4 or 8 days respectively. Sixty 4-day-old nymphs and 60 8-day-old adults were collected, immediately frozen in liquid nitrogen, and stored at −80⋅C for RNA extraction. Three independent biological replicates were performed for transcriptome sequencing analysis.

Total RNA was extracted with TRIzol Reagent (Invitrogen, Carlsbad, CA, USA). RNase-Free DNase (Qiagen, Valencia, CA, USA) was added to remove residual DNA. Samples were then further purified using RNeasy Mini Kit (Qiagen) according to the manufacturer's instructions. Purified total RNA samples were quantified using a NanoDrop ND-1000 spectrophotometer (NanoDrop Technologies, Wilmington, DE, USA) and qualified by Agilent Bioanalyzer (Agilent Technologies, Palo Alto, CA, USA). Transcriptome sequencing was performed on an Illumina HiSeq 2500 platform with 125-nucleotide (nt) paired-end reads at Texas A&M AgriLife Genomics and Bioinformatics Services (College Station, TX, USA).

### Sequence assembly and annotation

After trimming the adaptor sequences and removing short or low-quality reads (>5% unknown nucleotides or more than 20% nts with >10% error rate), the processed reads were assembled using Trinity software (Trinity Software, Inc., Plymouth, NH, USA) and clustered with TGICL Clustering tools (The Institute for Genomic Research, Rockville, MD, USA) (Pertea et al., 2003; Grabherr et al., 2011). The publically available databases, NCBI non-redundant (Nr), NCBI non-redundant nucleotide (Nt), Kyoto Encyclopedia of Genes and Genomes (KEGG), and Cluster of Orthologous Groups of proteins (COG) were used to perform BLAST analyses to annotate the functions of these assembled unigenes (*E*-value cutoff of 10^−5^). Blast2GO software (http://www.geneontology.org) was used for gene ontology (GO) annotations (Conesa et al., 2005).

### Differential gene expression and RT-qPCR confirmation

Genes differentially expressed between nymphs and adults were identified based on Fragments Per Kilobase per Million mapped reads (FPKM) values, which adjusts the number of fragments mapped to a transcript by the total number of fragments mapped to all unigenes and the length of the transcript (Mortazavi et al., 2008; Ji et al., 2013). The false discovery rate (FDR) was used for the *P*-values in multiple tests and analyses. A FDR ≤ 0.001 and an absolute value of the log_2_ ratio ≥ 1 provided significance threshold for gene expression differences.

To validate the FPKM analysis, expression of 20 selected genes were measured in nymphs and adults by RT-qPCR. For each total RNA sample, 2 μg RNA was used to synthesize cDNAs with random hexamer primers (Invitrogen) and M-MuLV reverse transcriptase (New England Biolabs, Beverly, MA, USA). qPCR reactions were performed using Power SYBR Green PCR Master Mix (Applied Biosystems, Foster City, CA, USA) according to the manufacturer's protocol and run on the CFX384^TM^ Real Time System (BioRad, Hercules, CA, USA). Dissociation curve analyses were performed to ensure amplification specificity. Mean fold change in gene expression was calculated as described previously (Chi et al., 2011). Primer sequences are provided in Table S1. The 18S rRNA gene of green peach aphid (Acc. No. AF487712.1) was amplified as the internal control.

### Functional analysis of differentially expressed unigenes

GO enrichment analysis was performed to recognize the main biological functions of differentially expressed unigenes. The hypergeometric test was performed to find significantly enriched GO terms in differentially expressed unigenes compared to the whole reference transcriptome background (Su et al., 2012; Ji et al., 2013). The *P*-value was calculated with the formula:

$$P\;=\;1\;-\sum_{i\;=\;0}^{m-1}\frac{\left(\begin{array}{c} M\\ i \end{array}\right)\left(\begin{array}{c} N-M\\ n-i \end{array}\right)}{\left(\begin{array}{c} N\\ n \end{array}\right)}$$

where N and n are defined as the number of genes in the transcriptome and differentially expressed genes with GO annotations, respectively. The variables *M* and *m* represent the gene number in the transcriptome annotated to a certain GO term and differentially expressed genes within the group (M-m ≥ 0), respectively. The calculated *P*-value was subjected to Bonferroni correction. GO terms with corrected *P*-value, i.e., *Q* < 0.05 were considered significantly enriched.

KEGG analyses were performed to identify significantly enriched pathways represented by differentially expressed unigenes. The hypergeometric test was used in a similar way to that for GO enrichment analysis and the terms with *Q* < 0.05 were determined as enriched pathways.

### Ka and Ks analyses

To predict CDS regions, unigenes were first aligned by BLAST analyses with *E*-value cutoff of 10^−5^ to public databases in the priority order of Nr, Swiss-Prot, KEGG, and COG. Coding regions with the best match in BLAST were considered to be the CDS. Unigenes unable to be aligned to any databases were scanned by ESTScan, which may predict some coding regions. The CDSs of pea aphid were predicted from the mRNA sequence data (https://www.aphidbase.com/aphidbase/content/download/3250/33670/file/aphidbase_2.1b_mRNA.fasta.bz2).

After filtering the redundant CDSs that may result from alternative splicing, predicted CDSs of the two aphid species were used to identify orthologous genes using OrthoMCL (Li et al., 2003). Only single-copy ortholog pairs longer than 150 bp were considered as putative orthologous gene pairs. Ka, Ks, and Ka/Ks-values were computed using the YN method implemented in the software KaKs Calculator Version 1.2 (Yang and Nielsen, 2000; Wang et al., 2011, 2014). As the sequencing errors were distributed among synonymous and non-synonymous sites at equal frequencies, they were not expected to strongly influence the results of analyses (Tiffin and Hahn, 2002; Wang et al., 2011, 2014).

## Results and discussion

### Illumina sequencing analysis and *de novo* assembly

High-throughput RNA-seq generated the most extensive current transcriptome for the green peach aphid. After quality checks, about 74.1, 74.0, and 74.5 million reads were obtained from the three replicates of nymphs and 74.6, 76.0, and 74.3 million reads from adults (Table 1). All reads were deposited in the NCBI Short Read Archive (SRA, the accession number SRP073458). The reads were assembled into 89,944, 85,416, and 82,810 contigs with mean lengths of 474, 502, and 460 nt for nymphs and 81,641, 78,710, and 87,354 contigs with mean lengths of 472, 484, and 464 nt for adults (Table 1). Using paired-end joining and gap-filling, these contigs were finally assembled into a total of 62,627 consensus sequences with a mean length of 1460 nt. GC contents were 39.00% for nymphs and 39.63% for adults, comparable to that of the pea aphid (38.80%) (Wang et al., 2014).

**Table 1:** **Summary of transcriptome parameters of green peach aphid nymphs and adults**.

	**Nymph**			**Adult**		
	**1^a^**	**2**	**3**	**1**	**2**	**3**
Number of processed reads	74,068,728	74,017,762	74,553,158	74,568,296	76,017,216	74,335,676
Number of contigs	89,944	85,416	82,810	81,641	78,710	87,354
Mean length of contigs (nt)	474	502	460	472	484	464
GC content (%)	39.11	38.89	38.06	39.58	39.69	39.44
Number of unigenes	61,186	55,776	60,271	53,928	52,829	57,758
Mean length of unigenes (nt)	1054	998	957	960	965	986

a *Values combined all independent biological replicates*.

### Functional annotation and classification of the assembled unigenes

Of the 62,627 unigenes, 33,543 were annotated by referencing to the Nr database (Table S2); 66.66% of the annotated sequences had very strong homology (*E* < 10^−60^), 12.02% showed strong homology (10^−60^ < *E* < 10^−30^) and the rest 21.32% showed homology (10^−30^ < *E* < 10^−5^) to known sequences. With respect to species, 92.30% of the unique annotated sequences matched to pea aphid, 1.45% to *Tribolium castaneum*, 0.49% to *Bombus impatiens*, and 0.41% to *Camponotus floridana*.

**Figure 1:**
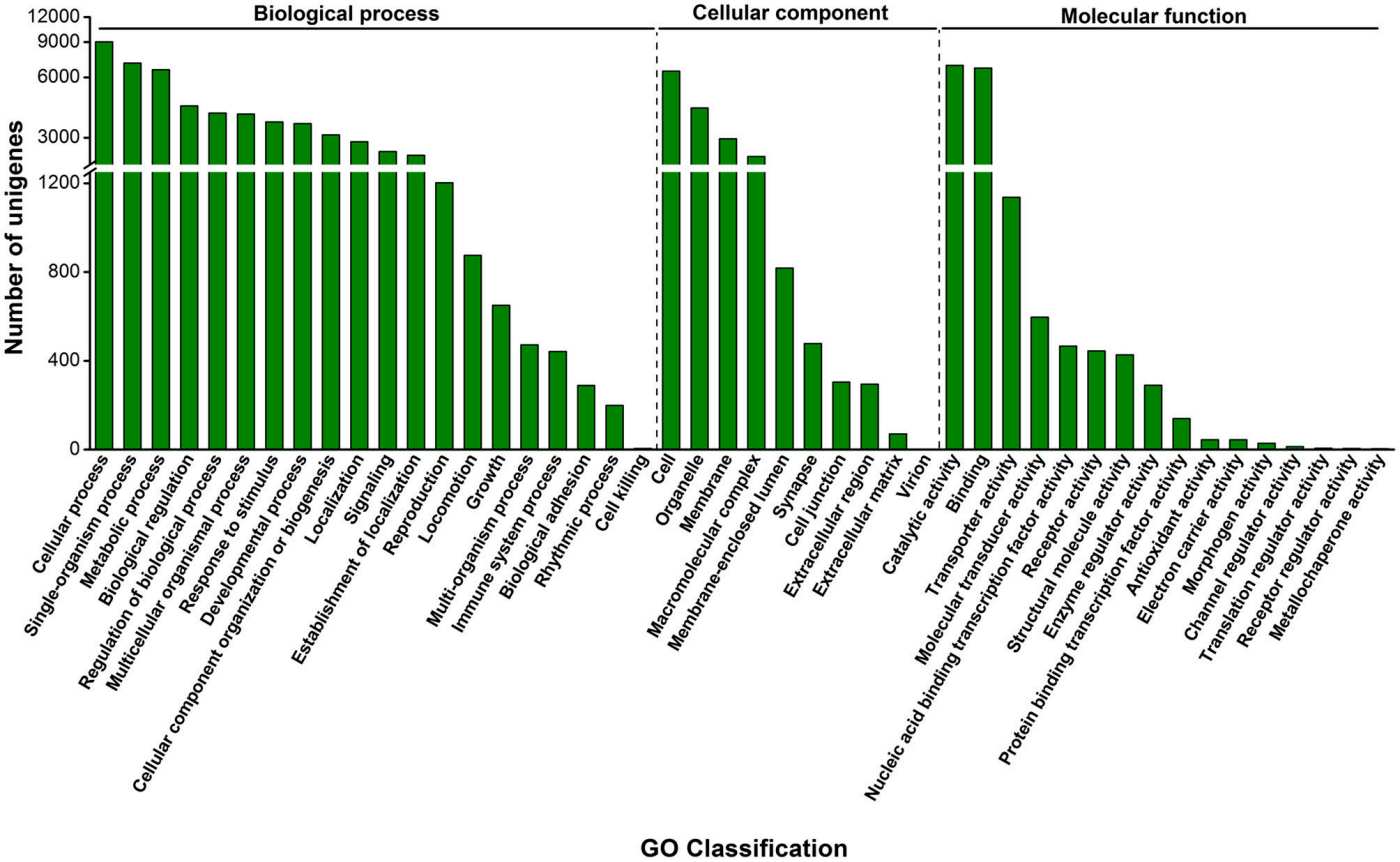
**Distribution of green peach aphid sequences by GO category**. GO classification includes three domains: biological process, cellular component, and molecular function. The y-axis shows the number of matching unigenes in a category.

GO assignments were used to classify the functions of the predicted unigenes; 14,260 sequences were categorized into 46 GO terms consisting of three domains: biological process, cellular component and molecular function (Figure 1). The most abundantly expressed genes in “biological process” were involved in cellular process (9028), single-organism process (7075), and metabolic process (6557). In “molecular function,” genes involved in catalytic (6894), binding (6678), and transporter (1137) activities were most abundantly expressed (Figure 1).

To better understand the biological pathways that are active in the green peach aphid, we mapped all sequences to the canonical reference pathways in the KEGG database. As a result, 23,695 sequences were assigned to 187 insect-related KEGG pathways (Table S3), with 3286 unigenes (15.47%) being involved in metabolic pathways. These annotations could be useful for further investigation of specific processes, functions and pathways.

### Comparison of gene expression profiles between nymphs and adults

**Figure 2:**
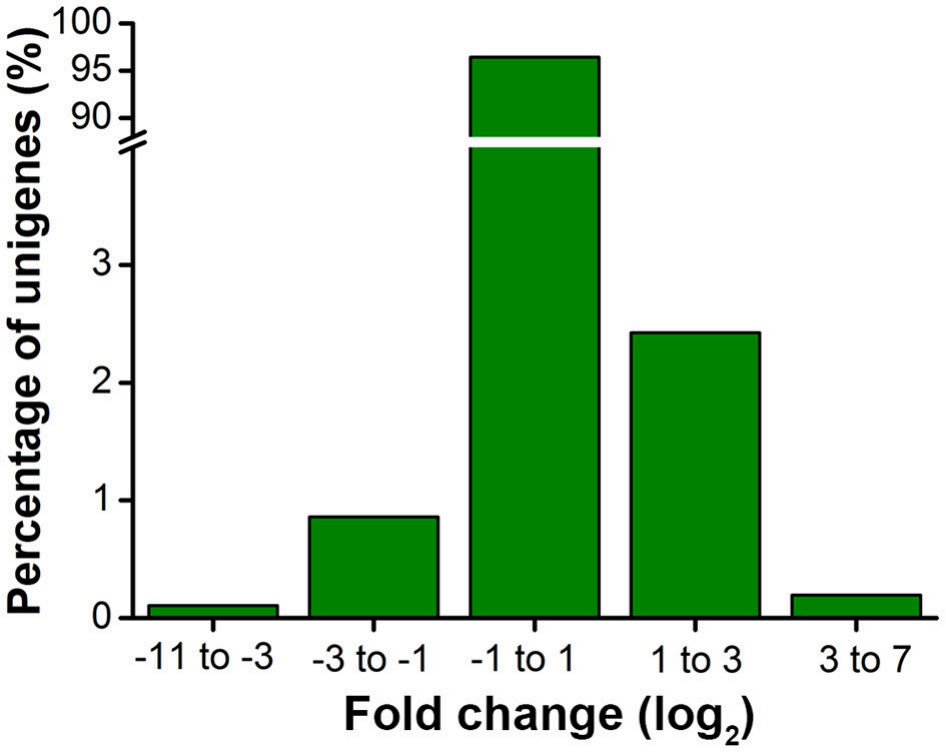
**Fold change distribution of green peach aphid unigenes between nymphs and adults**. The x-axis shows the fold change (log_2_ ratio) of gene expression in nymphs compared to adults. |Log_2_| values of 2244 unigenes are higher than 1, indicating potential importance during developmental transition.

When different developmental stages were compared, 1639 genes showed higher expression in nymphs and 605 higher in adults (Figure 2, Table S4). We performed RT-qPCR on selected genes to validate these gene expression data. Of the 20 selected genes, 18 were in agreement with RNA-seq results, suggesting good quality of transcriptomic analysis (Table S5). To gain insight into the major biological pathways represented by the differentially expressed genes, 21 enriched insect-related pathways (*Q* < 0.05) were identified using the hypergeometric test (Table 2); 14 were associated with “metabolism” and 3 with “digestive system,” suggesting differential metabolic and digestive activities between nymphs and adults (Banks and Macaulay, 1965; Randolph et al., 1975). The most enriched pathway being “metabolism of xenobiotics by cytochrome P450” is intriguing because it may reflect developmental stage-specific interaction with the host plant. Presumably, nymphal, and adult aphids ingest different amounts of allelochemicals, given that more detoxification genes, e.g., 16 of the 23 differential P450 genes, and all differential esterase (6) and GST (1) genes, were expressed in higher abundance in adults (Table 3). Developmental stage-dependent variations in expression patterns have often been observed in the detoxification genes (Harrison et al., 2001; Strode et al., 2006; Yang et al., 2013). High expression of *CYP321B1* is detected in the late larval stage of tobacco cutworm (*Spodoptera litura*) (Wang et al., 2016). In *B. tabaci*, relatively high expression of *CYP6CM1* is found in adults, correlating with the observation that specific resistance to neonicotinoid imidacloprid is largely restricted to adults (Nauen et al., 2008; Jones et al., 2011). Similarly, high expression of *CYP6P9* in adults of *Anopheles funestus*, but not in larvae, explains the adult resistance (Amenya et al., 2008). In a pyrethroid resistant strain of *Anopheles gambiae, CYP6Z1* is expressed in adults but undetectable in larvae or pupae (Nikou et al., 2003). Direct correlation between expression levels of detoxification genes at different developmental stages and resistance to pesticides is also exemplified by the beet webworm (*Pyrausta sticticalis*) (Leonova and Slynko, 2004) and citrus red mite (*Panonychus citri*) (Liao et al., 2013; Zhang K. et al., 2013). Banks and Macaulay (1965) reported that adult aphids have higher food consumption than nymphs. Ahmad (1982) stated that increased amounts of dietary allelochemicals due to increased food consumption may explain elevated P450-mediated metabolic activity. In parallel, green peach aphid adults likely ingest more plant materials, thus more allelochemicals from host plants, necessitating higher detoxification capacity.

**Table 2:** **Significantly enriched insect-related KEGG pathways represented by the genes differentially expressed between nymphs and adults**.

**Pathway**	***Q*-value**
Metabolism of xenobiotics by cytochrome P450	2.09 × 10^−7^
Steroid hormone biosynthesis	1.14 × 10^−6^
Retinol metabolism	1.34 × 10^−6^
Pentose and glucuronate interconversions	2.76 × 10^−6^
Ascorbate and aldarate metabolism	9.68 × 10^−6^
Glycine, serine and threonine metabolism	9.68 × 10^−6^
Circadian rhythm	2.81 × 10^−5^
Pentose phosphate pathway	1.92 × 10^−4^
Tyrosine metabolism	2.51 × 10^−4^
Glycerophospholipid metabolism	5.00 × 10^−4^
Glycerolipid metabolism	1.59 × 10^−3^
Starch and sucrose metabolism	2.93 × 10^−3^
Notch signaling pathway	6.75 × 10^−3^
Other types of O-glycan biosynthesis	7.06 × 10^−3^
RNA polymerase	7.09 × 10^−3^
Fat digestion and absorption	7.09 × 10^−3^
Insect hormone biosynthesis	1.99 × 10^−2^
Valine, leucine and isoleucine biosynthesis	2.45 × 10^−2^
Protein digestion and absorption	2.67 × 10^−2^
Vitamin digestion and absorption	3.92 × 10^−2^
Dorso-ventral axis formation	4.00 × 10^−2^

**Table 3:** **Differentially expressed detoxification and cuticle formation-related genes in adult and nymph**.

**Gene ID**	Fold change (log_2_)^*^	**Nr-annotation**
**DETOXIFICATION GENES UP-REGULATED IN ADULT**		
*Unigene28862*	7.29	Cytochrome P450 4g15-like
*Unigene5938*	5.20	Cytochrome P450 4g15-like
*Unigene38834*	4.64	Cytochrome P450 4C1-like
*Unigene12004*	3.09	Cytochrome P450 6a13-like
*CL1335.Contig8*	2.37	Cytochrome P450
*Unigene21970*	2.37	Cytochrome P450 6a13-like
*CL2142.Contig1*	2.13	Cytochrome P450 18a1-like
*Unigene8797*	2.03	Cytochrome P450 6a14-like
*Unigene13485*	1.73	Cytochrome P450 4C1-like
*Unigene17164*	1.65	Cytochrome P450 6j1-like
*CL4129.Contig2*	1.52	Cytochrome P450 4C1-like
*Unigene18192*	1.35	Cytochrome P450 18a1-like
*CL2142.Contig2*	1.31	Cytochrome P450 18a1-like
*Unigene30119*	1.23	Cytochrome P450 6a2-like
*CL1335.Contig7*	1.12	Cytochrome P450 6a13-like
*Unigene8106*	1.03	Cytochrome P450 4g15-like
*Unigene11947*	2.98	Esterase E4-like
*CL2237.Contig3*	1.97	Esterase FE4-like
*Unigene14425*	1.89	Esterase FE4-like
*Unigene30909*	1.60	Esterase FE4-like
*CL2237.Contig6*	1.28	Esterase FE4-like
*CL1600.Contig6*	1.14	Carboxylesterase-6-like
*Unigene8449*	1.07	Glutathione S-transferase D4-like
**DETOXIFICATION GENES UP-REGULATED IN NYMPH**		
*CL27.Contig6*	−4.63	Cytochrome P450 4C1-like
*Unigene12432*	−3.88	Cytochrome P450 4C1-like
*CL27.Contig7*	−3.41	Cytochrome P450 4C1-like
*CL1617.Contig5*	−2.30	Cytochrome P450 6a14-like
*CL3489.Contig2*	−1.83	Cytochrome P450 4C1-like
*Unigene13770*	−1.24	Cytochrome P450 6k1-like
*Unigene24402*	−1.20	Cytochrome P450 6k1-like
**CUTICLE FORMATION-RELATED GENES UP-REGULATED IN ADULT**		
*CL2631.Contig2*	2.57	Cuticle protein-like precursor
*Unigene31315*	2.32	RR1 cuticle protein 5
**CUTICLE FORMATION-RELATED GENES UP-REGULATED IN NYMPH**		
*Unigene2111*	−4.53	Cuticle protein
*CL4114.Contig1*	−4.17	Cuticular protein-like precursor
*Unigene17916*	−4.16	Cuticular protein 11 precursor
*Unigene16021*	−3.72	Cuticular protein 11 precursor
*Unigene7580*	−3.69	Cuticular protein-like precursor
*Unigene27924*	−3.53	Cuticular protein 11 precursor
*Unigene25191*	−3.45	Cuticular protein 16 precursor
*CL4114.Contig2*	−3.45	Cuticular protein-like precursor
*CL5082.Contig1*	−3.15	Cuticular protein 21
*Unigene4987*	−2.93	Cuticular protein 22 precursor
*CL5082.Contig2*	−2.85	Cuticular protein 21
*Unigene21389*	−2.71	Cuticular protein 21
*CL6036.Contig9*	−2.58	Cuticular protein 28 precursor
*Unigene21306*	−2.45	Cuticular protein 22 precursor
*Unigene24136*	−2.36	Cuticular protein 62 precursor
*Unigene13474*	−2.30	Cuticular protein 22 precursor
*Unigene13465*	−2.25	Cuticular protein 28 precursor
*CL5767.Contig2*	−2.21	Cuticular protein CPG12-like precursor
*Unigene13461*	−2.19	Cuticle protein-like
*Unigene9175*	−2.17	Cuticular protein 23 precursor
*Unigene24655*	−1.99	Cuticular protein 47 precursor
*Unigene13436*	−1.91	Cuticular protein 9 precursor
*Unigene13449*	−1.89	Cuticular protein 28 precursor
*Unigene13417*	−1.88	Cuticular protein 9 precursor
*CL1419.Contig2*	−1.87	Cuticular protein 15 precursor
*Unigene8362*	−1.72	Cuticular protein precursor
*Unigene13478*	−1.72	Cuticular protein 1 precursor
*Unigene4947*	−1.71	Cuticular protein 47 precursor
*Unigene11489*	−1.71	RR1 cuticle protein 7 precursor
*Unigene13482*	−1.70	Cuticular protein precursor
*Unigene10882*	−1.70	Cuticular protein 60 precursor
*Unigene11447*	−1.68	Cuticular protein CPG12-like precursor
*Unigene17255*	−1.67	Cuticular protein 20 precursor
*CL4704.Contig1*	−1.66	Cuticular protein 57 precursor
*Unigene13459*	−1.65	Cuticular protein 37 precursor
*Unigene13431*	−1.65	Cuticular protein 1 precursor
*Unigene13477*	−1.63	Cuticular protein 16 precursor
*Unigene13435*	−1.63	Cuticular protein 16 precursor
*Unigene13480*	−1.60	Cuticular protein 9 precursor
*Unigene13432*	−1.59	Cuticular protein 45 precursor
*Unigene13457*	−1.58	Cuticular protein 16 precursor
*Unigene11389*	−1.58	Cuticular protein CPG12-like precursor
*CL1419.Contig4*	−1.58	Cuticular protein 15 precursor
*CL5117.Contig1*	−1.56	RR2 cuticle protein 2
*Unigene13440*	−1.55	Cuticular protein 45 precursor
*Unigene13416*	−1.55	Cuticular protein 1 precursor
*Unigene13481*	−1.53	Cuticular protein precursor
*Unigene13443*	−1.53	Cuticular protein 37 precursor
*CL1419.Contig1*	−1.50	Cuticular protein 15 precursor
*Unigene13479*	−1.47	Cuticular protein 28 precursor
*Unigene13452*	−1.46	Cuticle protein-like
*CL6036.Contig5*	−1.45	Cuticular protein 28 precursor
*Unigene13484*	−1.45	Cuticular protein 1 precursor
*Unigene31055*	−1.42	Cuticular protein 48
*CL1419.Contig3*	−1.41	Cuticular protein 15 precursor
*Unigene21674*	−1.41	Cuticular protein 52 precursor
*Unigene31334*	−1.40	Cuticular protein 20 precursor
*Unigene13420*	−1.39	Cuticular protein 37 precursor
*Unigene14603*	−1.37	Cuticular protein 28 precursor
*Unigene13424*	−1.35	Cuticular protein 16 precursor
*Unigene14604*	−1.34	Cuticular protein 28 precursor
*Unigene7739*	−1.32	Cuticular protein analogous to peritrophins 3-D1 precursor
*Unigene13438*	−1.26	Cuticular protein 28 precursor
*CL6036.Contig11*	−1.26	Cuticular protein 28 precursor
*CL6036.Contig6*	−1.24	Cuticular protein 28 precursor
*Unigene24750*	−1.23	Cuticle protein precursor
*Unigene13418*	−1.21	Cuticular protein 45 precursor
*CL6036.Contig10*	−1.18	Cuticular protein 28 precursor
*Unigene554*	−1.17	Cuticular protein 31 precursor
*Unigene14112*	−1.14	Cuticular protein 30 precursor
*Unigene13441*	−1.13	Cuticular protein 9 precursor
*Unigene8067*	−1.09	Cuticular protein 68 precursor
*Unigene13475*	−1.09	Cuticular protein 28 precursor
*Unigene13426*	−1.08	Cuticular protein precursor
*Unigene31278*	−1.07	RR1 cuticle protein 1
*CL6048.Contig2*	−1.05	Cuticular protein precursor
*Unigene1224*	−1.04	Cuticle protein-like
*Unigene13467*	−1.03	Cuticular protein 1 precursor
*Unigene24090*	−1.01	Cuticular protein 58 precursor
*Unigene5077*	−1.55	Ecdysis triggering hormone

* *Log_2_ (FPKM-value in adult/ FPKM-value in nymph)*.

**Figure 3:**
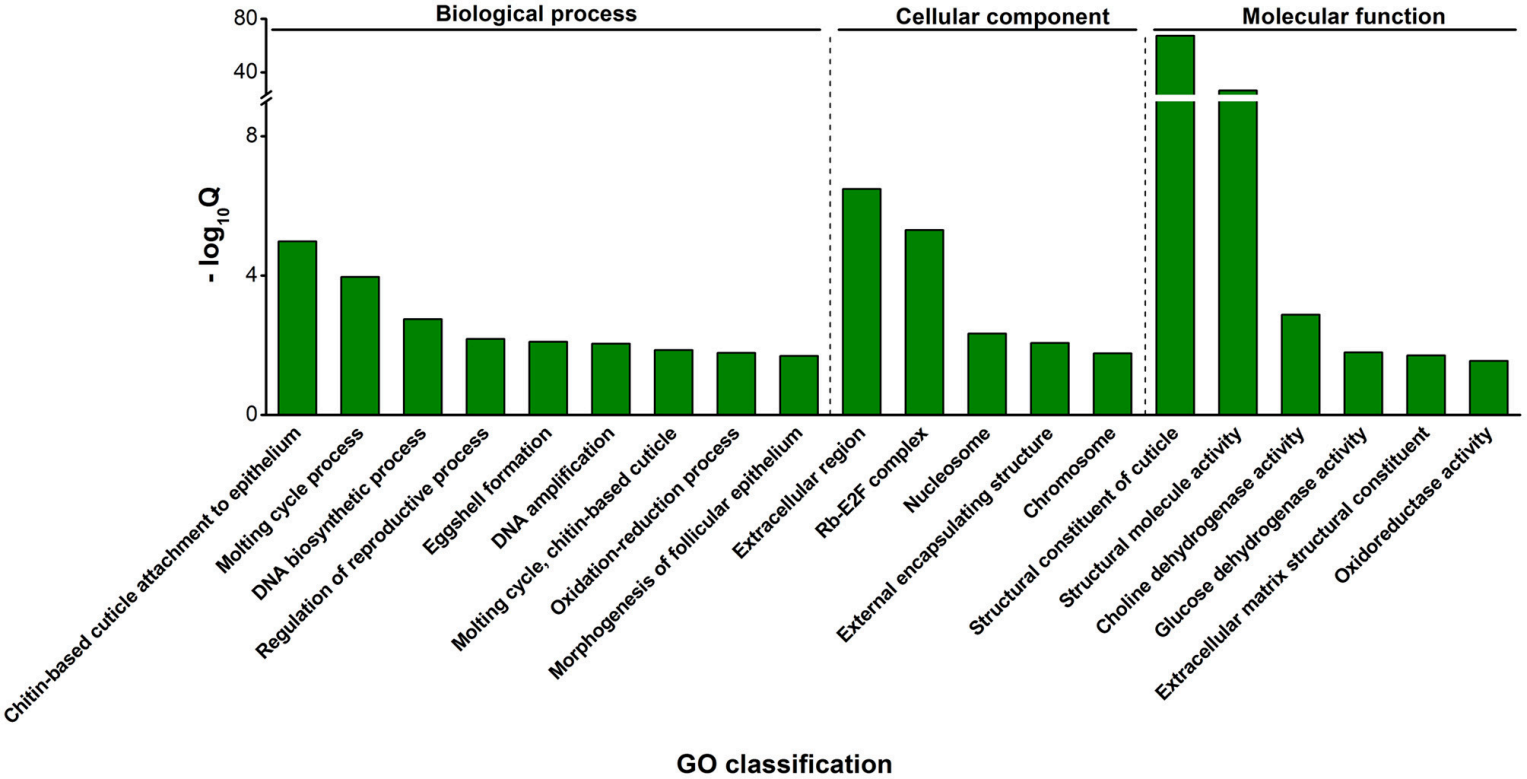
**Significantly enriched GO categories among the differentially expressed genes between nymphs and adults**. GO categories with *Q* < 0.05 were considered significantly enriched. Classification consists of three domains: biological process, cellular component and molecular function. The y-axis shows the value of −log_10_Q of the category. The GO term with highest −log_10_Q was determined the most significant enrichment.

The differentially expressed genes were also assigned to 20 GO enriched functional groups; ontology distributions are shown in Figure 3. Enriched in the “biological process” and “molecular function” include cuticle formation-related groups such as “structural constituent of cuticle,” “chitin-based cuticle attachment to epithelium” and “molting cycle, chitin-based cuticle.” The insect cuticle, composed of chitin and cuticle proteins, not only supports and maintains the physical structure, but also serves as a natural barrier against adverse external impacts (Andersen et al., 1995). Cuticle protein comparisons among insects at different developmental stages show that, rather than being an inert structure, the insect cuticle is developmentally modified (Chihara et al., 1982; Dombrovsky et al., 2003). Consistent with these findings, among the 81 differentially expressed transcripts of cuticular proteins and their precursors we detected, 79 were highly expressed in nymphs (Table 3). Insects of this developmental stage repeatedly shed their cuticles and replace them with new layers, thus their cuticle biosynthesis is likely more active. No doubt, hormones play an essential role in insect ecdysis. Enrichment of the “steroid hormone biosynthesis” pathway among the differential genes (Table 2) supports this notion. The major steroid hormone ecdysone plays an essential role in larval ecdysis, a process mediated by hormones, such as ecdysone and ecdysis triggering hormone (ETH) (Robbins et al., 1968; Ewer et al., 1997). Interestingly, the ETH-encoding gene *Unigene5077* was highly expressed in green peach aphid nymphs (Table 3).

### Transcriptomic divergences between green peach aphid and pea aphid

Transcriptome comparisons of different aphid species could provide useful information in understanding transcriptome evolution and the genetic factors underlying the biological divergence of these species. To identify genes specific to green peach aphid (relative to pea aphid), we compared the transcriptome we obtained in this study with publically available mRNA sequence data of pea aphid. tBLASTx identified homologous pea aphid mRNAs for 41,912 of our unigenes, leaving 20,595 having no hits. After removing sequences shorter than 250 bp (too short to be translated into polypeptides meaningful for comparisons) and BLASTn hits from pea aphid mRNAs and Nt databases, the remaining 13,752 were considered green peach aphid-specific unigenes under the rearing conditions described (Table S6).

Arabidopsis was selected as our host plant because it is readily consumed by green peach aphid. Its short life cycle, abundant genetic resources and well developed RNAi technique (Ramsey et al., 2007; Pitino et al., 2011; Bhatia et al., 2012; Elzinga et al., 2014) can greatly facilitate our more in-depth studies of candidate genes derived from the current study. One caveat however, is that choice of hosts may impact aphid gene expression. Few studies have been conducted to compare transcriptome profiles of the same insect species feeding on different host plants, but some information is available on differential gene expression of insect populations reared on different varieties/lines of the same host plant species (Ji et al., 2013; Bansal et al., 2014; Yu et al., 2014). It appears that the vast majority of genes are present (but likely varied in expression level) among different insect populations, and that genes solely expressed in one population are rare. Whether this observation can be extended to insect populations feeding on different host plant species is yet to be determined.

**Figure 4:**
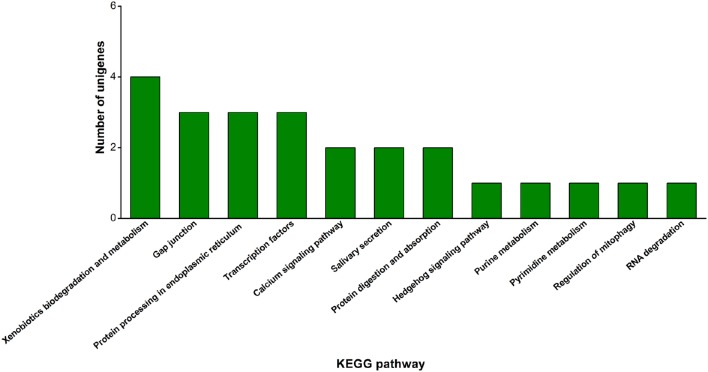
**Insect-related KEGG pathway classifications of green peach aphid-specific unigenes**.

The Nr, Nt, Swiss-Prot, COG, KEGG, and GO annotations of green peach aphid-specific unigenes were then performed (Table S6). Only 4.52% were predicted to have defined functions (Table 4), and functions of the remaining sequences need further study in the future. Likewise, KEGG classification identified only 30 unigenes, the most predominant group being “xenobiotics biodegradation and metabolism” (13.33%) (Figure 4). This finding correlates well with the fact that green peach aphids feed on a wider variety of plant species, and may have to encounter more types of toxic plant metabolites than pea aphids.

**Table 4:** **Annotations of green peach aphid-specific unigenes**.

**Public database**	**Number of annotated unigenes**	**Percentage (%)**
Nr	85	0.62
Nt	562	4.09
Swiss-Prot	31	0.23
COG	9	0.07
KEGG	30	0.22
GO	10	0.07

### Ka and Ks analysis between green peach aphid and pea aphid

Contrasting with the above analysis where the focus was on genes unique to green peach aphid, here we concentrated on single-copy orthologous genes between the two aphids. From the 33,963 green peach aphid CDSs (mean length, 1275 bp) derived from our RNA-seq, 3989 that had one-to-one orthologs in pea aphid CDSs were identified, and 3824 contained both substitution types, from which Ka/Ks ratios were calculated (Table S7).

The Ka/Ks ratio provides information about the evolutionary forces operating on a particular coding gene and has been widely used to measure the intensity and mode of selection; Ka/Ks = 1 indicates a neutral evolution; Ka/Ks < 1 suggests that nonsynonymous mutations are deleterious and purged from the population; Ka/Ks>1 indicates that nonsynonymous amino acid substitutions offer fitness advantages and are fixed in the population at a higher rate than synonymous substitutions (Hurst, 2002). However, this cutoff value for positive selection has recently been adjusted to 0.5 by Swanson et al. (2004). They found that 15 of 16 genes with 0.5 < Ka/Ks < 1 showed statistical evidence for adaptive evolution (Swanson et al., 2004). Since then, this new value has been adopted for “positive selection” determination in many studies (Kelleher et al., 2007; Elmer et al., 2010; Yang et al., 2012; Zhang L. et al., 2013; Ren et al., 2014; Cheng et al., 2015; Mu et al., 2015; He et al., 2016; Pereira et al., 2016). In our study, a total of 24 pairs of orthologs had a Ka/Ks ratio greater than 1, and 124 had a Ka/Ks ratio between 0.5 and 1 (Table S8).

Relative to the earlier study by Ollivier et al. (2010), our CDS construction is more complete than that of EST-based (33,963 CDSs, 1275 bp mean length vs. 6652 CDSs, 667 bp mean length), due to improvements in sequencing technology. Nevertheless, some putative orthologs under positive selection were identified by both studies, such as C002 (Table S8). Other genes related to insect-plant interactions include those encoding mucins (Ka/Ks = 1.09 and 0.94), the essential components of peritrophic matrix. Fast-evolving mucin proteins presumably contribute to aphid adaptation to different dietary pro-oxidants, phenolic, and lipophilic xenobiotics associated with their respective host plants (Hiraishi et al., 1991; Felton and Summers, 1995; Barbehenn, 1999, 2001; Barbehenn and Stannard, 2004; Hegedus et al., 2009). Likewise, the homolog of salivary protein gene *Me17* (Ka/Ks = 0.69), identified in multiple aphid species with dissimilar plant host ranges, is thought to play important roles as the effector in suppressing defense responses in different host plants and in promoting aphid colonization (Atamian et al., 2013; Pitino and Hogenhout, 2013; Elzinga et al., 2014). Nicotinic acetylcholine receptors (nAChR) in insects are often the target sites for naturally occurring and synthetic insecticides (Millar and Denholm, 2007; Bass et al., 2011). A high mutation rate in the nAChR β-2 subunit (Ka/Ks = 0.55) could help green peach aphids adapt to tobacco and become resistant to nicotine, as is the strain used in this study (Devine et al., 1996; Nauen et al., 1996). Another interesting ortholog pair encode odorant-binding protein 10 (OBP10) (Ka/Ks = 0.52). Nucleotide and amino acid sequence comparisons between the two species indicated that all substitutions occurred in the predicted mature protein region, and 19 of the 62 substitutions resulted in 12 hydrophilic and hydrophobic amino acid conversions (Figure S1). Sun et al. (2012) observed that the two aphid species showed similar as well as dissimilar behavioral responses to certain tested odors. Presumably, fast evolution in OBPs could contribute to the change in their binding activity, which in turn could facilitate host shift or impact host range (Matsuo et al., 2007; Sun et al., 2012).

## Conclusions

Our RNA-seq data have increased molecular resources available for the green peach aphid, a major agricultural pest as well as a biological model for insect-plant interaction studies. The transcriptomic analyses have deepened our understanding of aphid development and aphid-plant interactions. Our results have also provided useful insight into the molecular mechanisms underlying the biological variations in aphids, especially in adaptation to different host plants.

## Author contributions

KZS, JF, and RJ conceived and designed the experiments. RJ performed the experiments. RJ, YW, YC, MZ, HZ, LZ, and KZS contributed to the transcriptome data analysis. RJ, JF, and KZS wrote the manuscript. All authors contributed to the discussion and approved the final manuscript.

## Funding

This research was supported by the National Natural Science Foundation of China (31501636), the China Postdoctoral Science Foundation (2014M551529), Jiangsu Agricultural Science and Technology Independent Innovation Fund (CX(15)1055), Texas A&M Genomics Seed Grant, Texas Invasive Ant Research and Management Seed Grant, and the USDA-AFRI grant (2014-67013-21781).

### Conflict of interest statement

The authors declare that the research was conducted in the absence of any commercial or financial relationships that could be construed as a potential conflict of interest.
