# Supplementary material for: Transcriptome Analysis of Green Peach Aphid (Myzus persicae): Insight into Developmental Regulation and Inter-Species Divergence
Source: Front Plant Sci. 2016 Oct 21;7:1562. doi: 10.3389/fpls.2016.01562 (PMC5072348; doi:10.3389/fpls.2016.01562)
Supplement: Supplementary file 9 [file Image1.PDF]

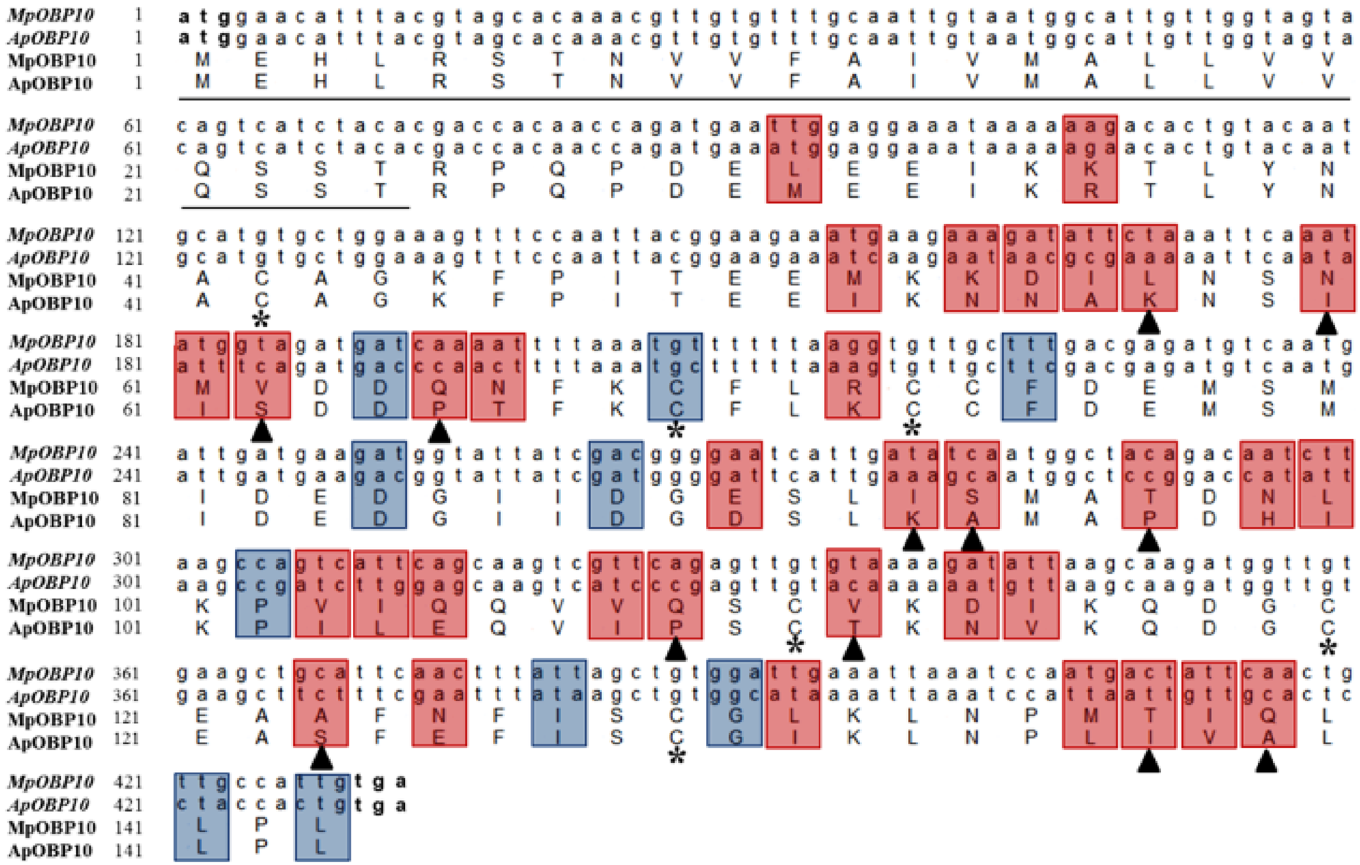

Figure S1. Sequence alignment of odorant-binding protein 10 (OBP10) ortholog between green peach aphid and pea aphid. Shown are the cDNA (lower cases) and the deduced amino acid (upper cases) sequences. Blue and red boxes indicate the synonymous and nonsynonymous substitutions, respectively. Arrow heads point to the substitutions that lead to hydrophilic and hydrophobic amino acid conversions. Asterisks mark the positions of six conserved cysteine residues present in all known insect OBPs. Predicted signal peptide is underlined. Start and stop codons are boldfaced.
